# Intensification of Type D Personality Traits and Coping Strategies of People Staying in Polish Penitentiary Institutions—Cross-Sectional Study

**DOI:** 10.3390/ijerph19042301

**Published:** 2022-02-17

**Authors:** Kinga Kołodziej, Anna Kurowska, Anna Majda

**Affiliations:** Laboratory of Theory and Fundamentals of Nursing, Institute of Nursing and Midwifery, Faculty of Health Sciences, Jagiellonian University Medical College, Michałowskiego 12 Street, 31-126 Cracow, Poland; anna2.kurowska@uj.edu.pl (A.K.); anna.majda@uj.edu.pl (A.M.)

**Keywords:** type D personality, coping strategies, stress, prisoners, mental health

## Abstract

The aim of this study was to determine the intensity of the occurrence of stress-prone personality traits (type D) and the strategies of coping with stress in a group of people staying in Polish penitentiary institutions. This study was conducted in two penitentiary units in Lesser Poland Voivodeship. Participants consisted of 152 prisoners. In this cross-sectional study, two standardized research tools—Mini-COPE Inventory for Measurement Coping with Stress and the Type-D Scale (DS14)—were used. The presence of a stress-prone personality (type D) among persons serving a prison sentence concerned 42.11% of the respondents and did not correlate with their sex and age. The convicted persons were more often guided by ‘negative affectivity’ than by ‘social inhibition’. People staying in penitentiary units most often used the strategy of ‘active coping’ and ‘planning’. Types of coping strategies used by the respondents depended on their sex and the presence of type D personal characteristics. Increasing people’s social awareness of the issues of stress experienced by prisoners can contribute to reducing the phenomenon of the marginalization and stigmatization of incarcerated people.

## 1. Introduction

There are 130 penitentiary units operating throughout Poland in fifteen District Inspectorates of the Prison Service [1]. In 2019, an average of 74,564 people were held in prisons and remand centers throughout Poland—3202 women and 71,362 men [2]. The incidence of mental health problems is higher in the prison population than in the general population [3,4]. The World Health Organization estimated that out of 9 million people staying in penitentiary institutions around the world, at least 1 million (11%) suffer from various types of mental disorders, the most common of which are depression and anxiety, which are the direct consequences of experiencing stress and inadequate coping [5,6].

Prison isolation is a stressful situation that results in the deprivation of needs in the field of, among others, personal autonomy, self-determination, and emotional contacts [7,8]. Persons staying in penitentiary units are forced to deal with excessive noise, adaptation to the rules prevailing in the institution, and sexual intimidation by fellow prisoners. According to the position of American scientists Mitchell Silverman and Manuel Vega, the prison is an unfamiliar environment for convicted person, to which he or she reacts with a culture shock [9]. A person who ends up in prison from the very beginning has to face a different reality from when free, adapt to many restrictions and perform assigned tasks. These situations are the most difficult for an individual in the initial phase of their stay in prison. The prisoners must get used to a change in environment, being constantly with the same people and a lack of privacy. If a person does not have personality traits that contribute to the acceptance of such conditions, then rebellion, negative behavior of the prisoner, conflict, and negative health consequences such as depressive states or mental disorders may occur, which may lead to suicide attempts [10,11].

In the 1990s, Dutch psychologist Johan Denollet introduced the concept of the type D personality (stress-prone personality), which contributes to the intensified experience of stress. It consists of two dimensions: ‘negative affectivity’ (worrying, irritation) and ‘social inhibition’ (unsociability, social imbalance), which are considered to be its permanent features [12]. Patients characterized by this type of personality are more likely to experience various difficulties in the recovery process—they more often report complaints about their somatic condition, experience anxiety and depression, and have low quality of life. Negative emotional states such as anxiety and depression affect their health indirectly. They act in a way that is harmful to their health, for example by engaging in risky behaviors (e.g., smoking, drug use, compulsive eating) [13]. People with this type of personality sacrifice their own emotional expression at the cost of social approval. Research shows that this pattern of behavior is a predictor of ischemic heart disease [14]. Previous studies show that the type D personality is also associated with the occurrence of infertility, cancers, gastrointestinal and duodenal ulcer disease, as well as skin and kidney diseases [15,16,17]. The above-mentioned disease entities are also mentioned as one of the most common chronic diseases among incarcerated people in old age [18]. 

Research shows that women are more likely to score higher for “negative affectivity”, while men have a higher score for “social inhibition”. This means that women tend to over-experience negative emotions more often, and men refrain from revealing them. It has been shown that elderly people, compared to young people, obtain higher scores for both D dimensions, similarly to cardiological patients compared to healthy people [12,16,19].

Type D personality influences coping strategies undertaken in times of stress. The research provides data indicating a positive relationship of both “negative affectivity” and “social inhibition” with the use of non-adaptive stress coping strategies, such as: “behavioral disengagement”, “venting”, “denial”, “self-distraction” and “substance use” [12,16,19]. The effectiveness of resolving a stressful situation is related to the appropriate adjustment of the coping process to the requirements of the situation. Moreover, it depends on factors related to the personality and environment of the individual [20]. Adequate primary and secondary assessment enables the selection of specific coping strategies [21]. Contact with informal structures of the ‘prison subculture’ and the characteristics of the environment prevailing in a penitentiary unit may influence the orientation of prisoners towards particular coping strategies. It has been proven that convicted persons who are able to accept the situation in which they find themselves and conduct self-reflection practices show disorders related to internalization in penitentiary institutions less frequently. Effective coping with stress is especially important in people imprisoned for the first time [22].

The activity aimed at coping with stressful events is referred to as ‘coping with stress’, and is now considered one of the most important components of stressful situations. Coping can be considered as a process, strategy or style [17,23]. The process of coping with stress constitutes the entirety of activities undertaken by a person in a specific stressful situation. It is complex, dynamic, and sometimes lasts for a long period of time [24]. 

According to the transactional concept of the American psychologists Richard Lazarus and Susan Folkman, coping with stress involves constant changes in cognitive and behavioral efforts aimed at mastering both internal and external requirements judged by the person to be onerous. According to the above theory, the goals of making such an effort include lowering unpleasant tension (regulatory function) and actions aimed at solving the problem (instrumental function). Both of them can be fulfilled simultaneously, interchangeably and are not mutually exclusive [25].

Coping styles, as defined by the Polish psychologist Irena Heszen-Niedojek, are a repertoire of coping strategies in stressful situations that are characteristic of an individual [26]. One of the first classifications of stress coping styles is the concept developed in 1987 by the psychologist Suzanne Miller. This distinguished two coping styles: focusing on the stressor and/or one’s own reaction to its operation, and focusing on distracting attention from the stressor and one’s own reactions [27]. 

In 1990, the psychologists Norman Endler and James Parker developed the transactional theory of stress, which follows the theories of Richard Lazarus and Susan Folkman and distinguishes three styles of coping: task-focused style, emotion-focused style, and avoidance-focused style. The first one is characterized by the control of the origin of stress. The person takes action to remove the stressor, plans, sets priorities, puts other things off and focuses on the stressful situation, and looks for support. The style focused on emotions concerns the exclusion of emotional tension that arises as a result of stress stimuli and the subjective assessment of a given situation as a threat to an individual. The avoidance-focused style is associated with reducing the undesirable effects of stressors. It concerns taking up activities unrelated to a given stressful situation, which are associated with distraction or not taking any action and waiting for the stressor to end its impact [28]. It has been proven that criminals often use referral techniques to avoid the stressor, which leads to negative consequences: isolation from society, substance abuse, and a lack of family involvement [29].

The American psychologist Charles C. Carver, based on the concept of Norman Endler and James Parker, developed a multidimensional theory describing groups of actions assigned to individual coping styles of the above-mentioned authors. To problem-focused styles, he assigned aspects such as active coping, seeking for instrumental social support, planning, restraint coping, and the suppression of competing activities. The style focused on emotion included positive reinterpretation, denial, seeking of emotional social support, acceptance, and turning to religion. In his opinion, the author also described “useless” coping strategies, which included venting of emotions, behavioral disengagement, and mental disengagement [30]. So far, there has been no clear consensus on which coping strategies are the most effective. Some studies show that problem-focused coping reduces emotional stress, while emotion-focused coping paradoxically increases it, while other authors have noted the opposite pattern [31]. The literature describes that Carver’s problem-focused style positively correlates with the task-focused styles of Endler and Parker. Both styles assume problem solving by recognizing different thoughts about the problem, making efforts to understand the situation, anticipating events, choosing the most appropriate solutions, planning and implementing the solution to the problem, and consistency in action. The opposite is the case with the avoidance-focused styles of Endler and Parker, where mental and behavioral withdrawal have been shown to be forms of non-constructive problem-solving and are related to the above-mentioned “useless coping strategies” described by Carver [30,31]. The influence of intrapsychic strategies has an impact on minimizing or maximizing the perceived stress—it can complicate problems, as well as restore or disturb the mental balance, which affects the quality of the mental state of an individual [32,33].

Studies conducted in the past have described the mechanisms of coping with stress among prisoners and the number and conditioning of various offenses in penitentiary units [34]. Moreover, their effectiveness was analyzed in connection with the recidivism phenomenon [6]. The analysis of available studies shows that both people staying in penitentiary institutions for the first time, as well as recidivists and juvenile prisoners, often choose coping strategies that are inadequate to the life situation, which in turn increases mental tension. 

The ineffective selection of a stress coping strategy causes the intensification of internal tension in prisoners, which may translate into increased stress and depressive symptoms, which is a currently big problem in prisons all over the world [35,36]. There is a need to explore the subject of the stress-prone personality (type D) among people staying in penitentiary units, because a shortage of articles has been noted in this regard. Research on the coping strategies among prisoners is sparse. Basic knowledge regarding the selection of strategies for coping with stress, as well as illustrating the scale of the intensity of the occurrence of type D personality traits in the prison population, may be the basis for exploring detailed, extensive issues related to the mental health of prisoners, which would also include sociodemographic issues related to prisoners and those related to the type of committed crime. In addition, the data obtained through the study can be used to develop the mental health policy of prisoners. Obtaining this type of information may affect the effectiveness of therapeutic interactions carried out in penitentiary units, reduce offenses committed by people in prison and, most importantly, improve the mental state of people staying in penitentiary institutions. Strengthening psychological and psychiatric care in prisons may condition the improvement of the functional state of prisoners, which in turn may increase the probability of adapting to social life after leaving the penitentiary facility. This paper is one of a series of articles presenting the outcomes of studies that were carried out in a group of people staying in Polish penitentiary institutions. In this article, we focused only on the type D Personality characteristics and the coping strategies. The main aim of this study was to determine the intensity of the occurrence of stressful personality characteristics (type D) and the strategies of coping with stress in a group of people staying in Polish penitentiary institutions. Moreover, attempts were made to examine whether there is a relationship between the studied variables and the gender and age of the respondents. Additionally, the relationship between the type D personality and coping strategies was assessed.

## 2. Materials and Methods

### 2.1. Research Design

The study was conducted in two penitentiary units in Lesser Poland Voivodeship, which belong to the Regional Inspectorate of the Prison Service in Cracow. The consent of the directors of these institutions was obtained prior to the start of research activities. The consent of the Prison Service was obtained for the collection of sociodemographic data on the age and sex of prisoners. It was assessed that obtaining detailed data on persons staying in penitentiary units (e.g., type of crime committed, length of stay in prisons) could violate the anonymity of people participating in the study, as well as creating the risk of their identification. It was a cross-sectional study and consisted of one stage—the completion of the research tools by participants who met the inclusion criteria: (1) absence of mental illness; (2) sentencing to imprisonment; and (3) informed consent to participate in this study. 

### 2.2. Ethical Consideration

Before starting the research activities, on 27 June 2019, the consent of the Bioethics Committee was obtained. The research was conducted in accordance with the principles of good scientific practice and the principles of the Helsinki Declaration [37,38].

### 2.3. Participants

The study group consisted of 152 people staying in penitentiary institutions—94 men (61.84%) and 58 women (38.16%) who were between 20 and 80 years old. The largest percentage of the respondents were people aged 31–40 years old—47 people (30.92%). The second largest group were people aged 20–30 years old—43 people (28.92%). The prisoners aged 41–50 years old totaled 35 people (23.03%). The respondents aged 51–60 years old constituted 17 people (11.18%). The least numerous groups were prisoners aged 61–70 years old—9 people (5.92%)—and 71–80—1 person (0.66%).

### 2.4. Instruments

#### 2.4.1. The Type-D Scale (DS14)

The Type-D Scale (DS14) was developed by Nina Ogińska-Bulik, Zygfryd Juczyński and Johan Denollet. This research tool was used to study the traits of a stress-prone personality (Type D personality). The scale consisted of 14 statements, 7 of which measured the tendency to feel negative emotions (‘negative affectivity’—NA), and the remaining 7 measured the tendency to refrain from expressing these emotions and behaviors related to them (‘social inhibition’—SH). Each of the statements was assessed on a five-point scale (0—‘false’; 1—‘rather false’; 2—‘hard to say’; 3—‘rather true’; 4—‘true’). The scores for ‘negative affectivity’ and ‘social inhibition’ were calculated separately, adding up the number of points in each subscale. Before summing up, in questions 1 and 3, the scoring was be changed according to the rule: 0 = 4; 1 = 3; 3 = 1; 4 = 0. The range for each dimension was 0 to 28 points. Obtaining at least 10 points in each subscale allowed the individual to qualify as a person with a stressful personality (Type D personality). The higher the number of points obtained, the greater the intensity of the features that make up a given dimension (NA or SH). All of the statements, except for the two belonging to the factor ‘social inhibition’, correlated above 0.50 with the overall score of the factor to which they belonged. Cronbach’s α coefficient was 0.86 for ‘negative affectivity’ and 0.84 for ‘social inhibition’. The reliability of the scale, assessed by the test–retest method after three months, was 0.76 for ‘negative affectivity’ and 0.73 for ‘social inhibition’. The criterion validity of the DS14 was established through correlation with other tools (e.g., NEO-FFI, PSS-10, GHQ-28, PANAS, FCZ-KT), and was found to be similar to type D dimensions [19].

#### 2.4.2. Mini-COPE Inventory for Measurement Coping with Stress

The Mini-COPE Inventory for Measurement Coping with Stress (Mini-COPE) was developed by Charles Carver, and adapted by Zygfryd Juczyński and Nina Ogińska-Bulik. This research tool was used for measuring a person’s coping behaviors (strategies of coping with stress). The Mini-COPE consisted of 28 statements that are part of 14 strategies (two statements in each strategy): ‘active coping,’ ‘planning,’ ‘positive reframing,’ ‘acceptance,’ ‘humor,’ ‘religion,’ ‘use of emotional support,’ ‘use of instrumental support,’ ‘self-distraction,’ ‘denial,’ ‘venting,’ ‘substance use,’ ‘behavioral disengagement,’ and ‘self-blame,’ [34]. The respondent reacted to a given statement by selecting the answer from a four-point scale (0—‘I hardly ever do it’; 1—‘I rarely do it’; 2—‘I frequently do it’; 3—‘I do it most of the time’). The results were calculated separately for each coping strategy by adding the points for the answers included in the strategy and dividing this sum by 2. The higher the score obtained in a given dimension, the more frequent use of the coping strategy. The internal consistency of the Polish version of Mini-COPE was established by examining 200 people aged 25–60. The half-life reliability was 0.86 (Guttman’s index 0.87). Consistency, assessed based on a group of 34 subjects after six weeks, was satisfactory for most scales, including the highest for “religion” (0.94), “use of substances” (0.82) and the lowest for “behavioral disengagement” (0.32). Diagnostic relevance was assessed by correlating the Mini-COPE results with the Mini-MAC scale, which was designed to study the strategies of coping with cancer [19].

### 2.5. Data Collection

A total of 250 sheets of scales were delivered to the facilities for the research. The study was conducted in semi-open and closed prison wards, for people without diagnosed mental illness, non-psychotic mental disorders, intellectual disability or during psychiatric diagnosis. The study was carried out with the help of the employees of the penitentiary institutions. Prison staff, who had previously been told in detail about the study, told the respondents about the purpose and course of the study. In addition, the employees of the units explained how to correctly fill in the sheets and addressed doubts during the study among people who agreed to participate in it. The participants were informed that they can withdraw at any stage of the study, which was voluntary and anonymous. Finally, 168 people agreed to participate in the study. After the incomplete sheets were deleted, 152 scale sheets were finally qualified for statistical analysis. 

### 2.6. Data Analysis

In this study, statistical methods enabling the development and interpretation of the results were used. During the analysis of the research material, the analysis of quantitative variables was used, calculating the mean, standard deviation, median, quartiles, minimum and maximum. During the comparison of the values of quantitative variables in two groups, a Mann–Whitney *U* test was used. Correlations between quantitative variables were analyzed using the Spearman rank correlation coefficient.

In the DS-14 questionnaire, the comparison of the values of the qualitative variables was performed using the chi-square test. The analysis of the influence of quantitative variables on the dichotomous variable was performed using the logistic regression method. Results were presented as odds ratios (OR) with a 95% confidence interval (CI).

A significance level of 0.05 was adopted in the analysis. This means that all *p* < 0.05 values were interpreted as showing significant correlation. The analysis was performed using the R statistical program (version 3.6.2) [39].

## 3. Results

### 3.1. Type D Personal Characteristics 

The statistical analysis can be found in Table 1 and it shows that 64 people (42.11%) had a type D personality, while 88 people (57.89%) had a different personality type. When assessing the dimensions of the stress-prone personality, it can be concluded that among the prisoners, a greater number of respondents demonstrated ‘negative affectivity’ (M = 12.64) than ‘social inhibition’ (M = 10.44). 

In Table 2 and Table 3, we presented that the occurrence of type D personality was not related to the gender and age of the incarcerated persons (*p* > 0.05). 

### 3.2. The Coping Strategies

People staying in penitentiary institutions used strategies such as ‘active coping’ (M = 2.28; SD = 0.62) and ‘planning’ (M = 2.10; SD = 0.74) the most often. Strategies such as ‘acceptance’ (M = 1.80; SD = 0.68), ‘self-distraction’ (M = 1.78; SD = 0.70), ‘positive reframing’ (M = 1.73; SD = 0.76), ‘use of instrumental support’ (M = 1.66; SD = 0.76), ‘use of emotional support’ (M = 1.62; SD = 0.85), ‘self-blame’ (M = 1.49; SD = 0.83), ‘venting’ (M = 1.27; SD = 0.77) and ‘denial’ (M = 1.05; SD = 0.91) were used sporadically. Persons serving prison sentences used strategies such as ‘humor’ (M = 0.96; SD = 0.81), ‘religion’ (M = 0.88; SD = 0.97), ‘behavioral disengagement’ (M = 0.81; SD = 0.81) and ‘substance use’ (M = 0.80; SD = 0.97) the least. Table 4 displays the means and standard deviations of all variables. 

The statistical analysis showed the significant relationship between the respondents’ gender and the type of coping strategies. Strategies such as ‘positive reframing’ (*p* = 0.014), ‘acceptance’ (*p* = 0.02), ‘humor’ (*p* = 0.01), ‘use of emotional support’ (*p* = 0.016), ‘use of instrumental support’ (*p* = 0.025) and ‘self-distraction’ (*p* = 0.008) were used more often by women. Table 5 displays the means and standard deviations of all variables. 

The type of coping strategy was not related to the prisoners’ age (*p* > 0.05). Table 6 presents all of the variables.

### 3.3. Relationship between Respondents’ Type D Personal Characteristics and Coping Strategies

Table 7 presents significant correlations between the type of respondents’ personality and types of coping strategies used by them. The frequency of using strategies such as ‘behavioral disengagement’ (*p* = 0.001) and ‘self-blame’ (*p* = 0.029) was significantly higher in a group of prisoners with type D personality. In a group of prisoners with a different personality type, strategies such as ‘active coping’ (*p* = 0.001), ‘planning’ (*p* = 0.001), ‘positive reframing’ (*p* = 0.028), ‘use of emotional support’ (*p* = 0.016) and ‘use of instrumental support’ (*p* = 0.008) were the most popular. Table 7presents all of the variables.

## 4. Discussion

The study presented in this article explores the issue of coping with stress and the intensity of the occurrence of a stress-prone personality (type D) among men and women serving prison sentences. Publications emphasize the negative impact of imprisonment on mental health of incarcerated persons [40]. Research indicates that people who stayed in penitentiary institutions in the past show a significantly higher probability of depression and mood disorders [41], which are direct consequences of experiencing stress [42].

The analysis of the available literature showed that there are few works dealing with the topic of coping with stress and the intensity of the occurrence of a stress-prone personality in the group of people staying in penitentiary institutions, which was the motivation for the authors to explore the above-mentioned issues. 

The research results presented in this paper show that the persons staying in penitentiary institutions used strategies of coping with stress aimed at active coping the most often, while they used strategies related to helplessness, a sense of humor and the use of religious practices the least. Moreover, strategies related to active coping, seeking support, sense of humor, the use of avoidance behaviors and the acceptance of the experienced difficult situation correlated with gender and were used by women more often. Skowroński and Talik [43] conducted a study in Polish penitentiary units in a group of 390 male using the COPE questionnaire. They found that prisoners used strategies related to active coping the most often, and strategies related to the feeling of helplessness the least frequently, which is consistent with the results of the presented research. It can be assumed that prisoners use constructive strategies during a problematic situation mostly, which is undoubtedly positive. In addition, the above-mentioned authors explored the issue of the relationship between the quality of life of prisoners and their coping strategies. The convicted persons displaying a high level of overall quality of life preferred to use the following strategies: ‘active coping’, ‘planning’, ‘use of emotional support’ and ‘positive reframing’, suggesting that a person who experiences life satisfaction chooses active attitude during coping more often. The functioning of a person in the realities of prison isolation is extremely difficult, and prevents them from making decisions about themselves, which is undoubtedly one of the hardest conditions. According to the analysis of the available literature on the subject, in situations related to the lack of a sense of control over one’s own life, avoidance strategies are more effective [28,44]. Prisoners with a low sense of overall quality of life used strategies such as ‘denial,’ ‘self-distraction,’ ‘behavioral disengagement,’ ‘substance use’ and ‘humor’ most often, which are mostly included in avoidance strategies [44].

The results of a study by Belay [45] carried out in a group of 172 incarcerated women detained in penitentiary units in Ethiopia indicate that respondents used strategies of coping related to the use of religious practices and active coping the most frequently. This is only partially consistent with the results of the study presented in this article, where women often used strategies aimed at active coping, but rarely those related to religiosity. In the Belay study [45], age was a factor that negatively correlated with drug use strategies, as well as with a focus on emotions; as the age increased, the use of these strategies was less frequent. Moreover, a high level of perceived stress positively correlated with the use of strategies related to helplessness, acceptance of the situation and concentration on emotions. It should be noted that in the present study, convicted persons with high levels of stress used strategies related to the feeling of helplessness and the use of avoidance behaviors more often, while focusing on active coping the least frequently. This allows us to assume that people in penitentiary units who experience strong stress feel helpless in the face of their life situation. The feeling of helplessness is a negative phenomenon associated with the occurrence of increased mental tension, which may lead to the occurrence of depressive disorders among prisoners and, consequently, to attempting suicide [35,46,47,48,49].

During the analysis of the literature, studies on the presence of type D personality in the group of people staying in penitentiary units were not found. The analysis of the collected research material showed that almost half of the prisoners (42.11%) had a type D personality, defined as a stress-prone personality. Its occurrence was not related to respondents’ gender and age. It can be assumed that people staying in penitentiary units showing stress-prone personality traits (e.g., social withdrawal, pessimistic approach to life, worrying) perceived their environment as stressful and threatening. As a consequence, this may lead to chronic stress, which in the future may result in the development of stress-related diseases, in particular those related to the circulatory system [13,16,17]. The scale of the phenomenon of type D personality among the surveyed prisoners is disturbing and emphasizes the importance of the problem and the need to implement solutions aimed at reducing the intensity of perceived stress, as well as learning how to deal with it effectively.

The type D personality has two dimensions: ‘negative affectivity’ and ‘social inhibition’. ‘Negative affectivity’ is linked to neuroticism and extraversion, pessimism, assessing events as highly threatening, experiencing strong anxiety, and in social contacts is associated with a feeling of confusion and shyness. However, the dimension of neuroticism is a broader construct—it encompasses not only the tendency to experience negative emotions, but also takes into account emotional lability. ‘Social inhibition’ is similar to introversion, and it is connected with little tendency to seek social support and low self-esteem [19]. In the present study, a greater number of people were guided by ‘negative affectivity’ rather than by ‘social inhibition’. A study by Mosaku et al. [50] conducted in a group of 406 people in a penitentiary unit in Nigeria showed that few of the prisoners were characterized by neuroticism, while people exhibiting features of extrovertism constituted a larger group. Due to the differences in the research tools used, which define different personality constructs, it is difficult to perform a reliable comparison of personality traits between the prisoners presented in the study by Mosaku et al. and the respondents participating in the presented own study.

The study presented in this article showed that prisoners with a stress-prone personality type used strategies related to helplessness. In addition, the prisoners who presented a personality type other than the stress-prone personality were focused on seeking support and active coping. It can be assumed that people with stress-related personality traits are not able to find a solution in a difficult situation, which is a negative thing, because it may intensify the development of various types of disorders related to mental tension. Leszko et al. [51], in their work on the search for a relationship between personality traits and coping strategies, in which the studied group consisted of 465 men staying in penitentiary institutions in Poland, showed that people manifesting neuroticism were oriented towards coping based on emotions, while using action-oriented strategies the least frequently. Moreover, emotional-oriented coping positively correlated with extrovertism [51]. Analyzing the results of the study presented in this paper and the results from other studies, it can be concluded that the in-depth knowledge of the personality traits of incarcerated people by employees of penitentiary institutions may be a key element in identifying appropriate strategies for coping with stress, which will facilitate directing prisoners to appropriate methods of coping with them. It is likely that detainees often choose a given strategy because they have not skillfully mastered other coping mechanisms during the development stage. In particular, this may apply to people with type D personality, for whom the closest environment is often perceived as a threat that increases helplessness.

Knowing the mental state of prisoners, which includes the level of perceived stress, strategies for coping with it, and undoubtedly the intensification of the characteristics of a given personality, it is possible to develop new therapeutic programs or estimate the needs of those already well-known in Polish penitentiary conditions. The literature has proven the high effectiveness of training (usually lasting several weeks), which is a combination of meditation and yoga-mindfulness-based stress reduction, which was met with great approval among people staying at penitentiary units, and which was aimed at reducing the perceived stress, the adequate selection of methods of coping with it, and reducing the severity of aggression [52]. Similarly, a 10-day Vipassana course was adopted, also involving the reduction in mental tension through meditation [53]. Among juvenile offenders, the Stress Management Training Program, lasting 2 weeks with a total of 10 sessions of 40 min each, was highly effective. It ensured positive behavioral changes in selecting strategies for coping with stress [54]. Each of the above-mentioned examples of training could be adapted to the Polish penitentiary reality, due to the low cost of conducting them, as well as their duration, ranging from several days to several weeks, which increases the probability that they would be completed entirely by prisoners. Based on the evidence from the above studies, it can be concluded that the use of certain training in prison conditions may be a safe method of improving the results related to the quality of mental health of people staying in penitentiary institutions; however, it is necessary to further explore the above-mentioned issues in order to verify the most effective therapeutic methods.

The presented study should be considered in terms of its strengths and limitations. The strength of this study was the exploration of issues related to a specific study group, which was persons serving a prison sentence. In addition, the analysis was carried out in relation to a subject that has so far been scarcely explored in the scientific literature. An additional advantage of this study was the use of a variety of standardized research tools. The limitation of this research was the cross-sectional nature of the study, which, compared to experimental studies or those in which randomization was applied, shows less scientific value. In addition, the only socio-demographic data collected were their gender and age, which makes it difficult to reliably compare our results with the material of other authors who described relationships related to, among other things, education, civil status and the length of prisoners’ sentence. This was due to the confidentiality of information relating to the detainees in penitentiary units. The size of the study group was small, which makes relating the results to the general population difficult as well.

## 5. Conclusions

(a). People serving prison sentences used strategies related to active coping the most often and strategies related to sense of humor, turning to religion and helplessness the least. A gender correlation was shown—women used strategies related to seeking support, religious practices, sense of humor, active coping, acceptance, and avoidance behaviors most often. There was no correlation between the type of strategies and the age of the respondents.
(b). Almost half of the detainees had a stress-prone personality (type D). This type of personality did not depend on the gender and age of the prisoners. The convicted persons were more often guided by ‘negative affectivity’ than by ‘social inhibition’.
(c). The respondents with a type D personality used coping strategies related to helplessness the most often. The prisoners with a different type of personality used strategies focused on active coping and seeking support the most often.
(d). Based on the evidence, it can be concluded that the use of certain training in prison conditions may be a safe method of improving the results related to the quality of mental health of people in penitentiary institutions. The results obtained in this study may be useful in order to further verify the orientation towards the styles of coping with stress in prisoners.

## Figures and Tables

**Table 1:** Dimensions of type D personality in the group of people staying in penitentiary institutions.

DS14 (The Type D Scale)	The Range of Points	X	SD	Me	Min	Max	Q1	Q3
Negative Affectivity	0–28	12.64	7.05	12	0	28	8	18
Social Inhibition	0–28	10.44	7.19	9	0	28	5	15

X—mean; SD—standard deviation; Me—median; Min—minimum; Max—maximum; Q1—first quartile; Q3—third quartile. Source: author’s own analysis.

**Table 2:** The correlation between type D personality and the gender of people staying in penitentiary institutions.

DS14 (The Type D Scale)	Gender		*p* *
	Male (n/%)	Female (n/%)	
Type D personality	42/44.68	22/37.93	*p* = 0.516
Different type of personality	52/55.32	36/62.07	

n—number of subjects; *p*—statistical value; * Chi-square test. Source: author’s own analysis.

**Table 3:** The correlation between type D personality and the age of people staying in penitentiary institutions.

Variable	OR	95% CI		*p*
Age	1.017	0.990	1.045	0.217

OR—odds ratio; CI—confidence interval; *p*—statistical value.

**Table 4:** Strategies used by people staying in penitentiary institutions.

Type of Strategy (Mini-COPE Inventory for Measurement Coping with Stress)	X	SD	Me	Min	Max	Q1	Q3
Active coping	2.28	0.62	2.50	0.50	3.00	2.00	2.62
Planning	2.10	0.74	2.00	0.00	3.00	2.00	2.50
Positive reframing	1.73	0.76	2.00	0.00	3.00	1.00	2.12
Acceptance	1.80	0.68	2.00	0.00	3.00	1.50	2.00
Humor	0.96	0.81	1.00	0.00	3.00	0.50	1.50
Religion	0.88	0.97	0.50	0.00	3.00	0.00	1.50
Use of emotional support	1.62	0.85	1.50	0.00	3.00	1.00	2.00
Use of instrumental support	1.66	0.76	1.50	0.00	3.00	1.00	2.00
Self-distraction	1.78	0.70	2.00	0.00	3.00	1.50	2.00
Denial	1.05	0.91	1.00	0.00	3.00	0.00	1.50
Venting	1.27	0.77	1.25	0.00	3.00	0.50	2.00
Substance use	0.80	0.97	0.50	0.00	3.00	0.00	1.50
Behavioral disengagement	0.81	0.81	0.50	0.00	3.00	0.00	1.50
Self-blame	1.49	0.83	1.50	0.00	3.00	1.00	2.00

X—mean; SD—standard deviation; Me—median; Min—minimum; Max—maximum; Q1—first quartile; Q3—third quartile. Source: author’s own analysis.

**Table 5:** The correlation between the types of coping strategies and the gender of people staying in penitentiary institutions.

Type of Strategy (Mini-COPE Inventory for Measurement Coping with Stress)		Gender		*p* *
		Male (N = 94)	Female (N = 58)	
Active coping	X ± SD	2.2 ± 0.63	2.4 ± 0.58	*p* = 0.066
	Me	2.5	2.5	
	quartiles	2.0–2.5	2.0–3.0	
Planning	X ± SD	1.99 ± 0.79	2.26 ± 0.63	*p* = 0.052
	Me	2.0	2.0	
	quartiles	1.5–2.5	2.0–3.0	
Positive reframing	X ± SD	1.62 ± 0.72	1.91 ± 0.79	*p* = 0.014
	Me	2.0	2.0	
	quartiles	1.0–2.0	1.5–2.5	
Acceptance	X ± SD	1.71 ± 0.67	1.96 ± 0.68	*p* = 0.02
	Me	1.5	2.0	
	quartiles	1.5–2.0	1.5–2.5	
Humor	X ± SD	0.85 ± 0.82	1.14 ± 0.77	*p* = 0.01
	Me	0.5	1.0	
	quartiles	0.0–1.0	0.62–1.5	
Religion	X ± SD	0.81 ± 0.93	0.99 ± 1.02	*p* = 0.316
	Me	0.5	1.0	
	quartiles	0.0–1.5	0.0–2.0	
Use of emotional support	X ± SD	1.48 ± 0.78	1.84 ± 0.93	*p* = 0.016
	Me	1.5	2.0	
	quartiles	1.0– 2.0	1.0–2.5	
Use of instrumental support	X ± SD	1.55 ± 0.71	1.84 ± 0.8	*p* = 0.025
	Me	1.5	2.0	
	quartiles	1.0–2.0	1.0–2.5	
Self-distraction	X ± SD	1.65 ± 0.71	1.98 ± 0.64	*p* = 0.008
	Me	1.5	2.0	
	quartiles	1.5–2.0	1.5–2.5	
Denial	X ± SD	0.97 ± 0.91	1.18 ± 0.9	*p* = 0.142
	Me	1.0	1.0	
	quartiles	0.0–1.5	0.5–1.5	
Venting	X ± SD	1.17 ± 0.73	1.42 ± 0.8	*p* = 0.083
	Me	1.0	1.5	
	quartiles	0.5–1.5	0.5–2.0	
Substance use	X ± SD	0.84 ± 0.99	0.74 ± 0.93	*p* = 0.568
	Me	0.5	0.25	
	quartiles	0.0–1.5	0.0–1.0	
Behavioral disengagement	X ± SD	0.77 ± 0.77	0.86 ± 0.87	*p* = 0.653
	Me	0.5	0.75	
	quartiles	0.0–1.5	0.0–1.5	
Self-blame	X ± SD	1.43 ± 0.79	1.59 ± 0.88	*p* = 0.263
	Me	1.5	1.5	
	quartiles	1.0–2.0	1.0–2.38	

N—number of subjects; X—mean; SD—standard deviation; Me—median; *p*—statistical value. * Mann–Whitney *U*-test. Source: author’s own analysis.

**Table 6:** The correlation between the types of coping strategies and the age of people staying in penitentiary institutions.

Type of Strategy (Mini-COPE Inventory for Measurement Coping with Stress)	Age
	Correlation (Spearman Rank Coefficient)
Active coping	r = 0, *p* = 1
Planning	r = 0.064, *p* = 0.431
Positive reframing	r = −0.068, *p* = 0.402
Acceptance	r = 0.069, *p* = 0.396
Humor	r = −0.13, *p* = 0.111
Religion	r = −0.015, *p* = 0.855
Use of emotional support	r = 0.004, *p* = 0.959
Use of instrumental support	r = −0.123, *p* = 0.133
Self-distraction	r = −0.035, *p* = 0.667
Denial	r = 0.039, *p* = 0.634
Venting	r = −0.057, *p* = 0.489
Substance use	r = −0.089, *p* = 0.274
Behavioral disengagement	r = −0.033, *p* = 0.684
Self-blame	r = 0.082, *p* = 0.317

r—Spearman rank coefficient; *p*—statistical value. Source: author’s own analysis.

**Table 7:** The correlation between the type of respondents’ personality and types of coping strategies used by people staying in penitentiary institutions.

Type of Strategy (Mini-COPE Inventory for Measurement Coping with Stress)		DS14 (The Type-D Scale)		*p* *
		The Type D Personality (n = 64)	Different Type of Personality (n = 88)	
Active coping	X ± SD	2.06 ± 0.68	2.43 ± 0.52	*p* = 0.001
	Me	2.0	2.5	
	quartiles	1.5–2.5	2.0–3.0	
Planning	X ± SD	1.86 ± 0.77	2.27 ± 0.67	*p* = 0.001
	Me	2.0	2.5	
	quartiles	1.5–2,5	2.0–3.0	
Positive reframing	X ± SD	1.57 ± 0.73	1.84 ± 0.76	*p* = 0.028
	Me	1.5	2.0	
	quartiles	1.0–2.0	1.5–2.5	
Acceptance	X ± SD	1.67 ± 0.75	1.9 ± 0.62	*p* = 0.079
	Me	1.75	2.0	
	quartiles	1.0–2.0	1.5–2.12	
Humor	X ± SD	1.08 ± 0.99	0.87 ± 0.63	*p* = 0.501
	Me	1.0	1.0	
	quartiles	0.0–2.0	0.5–1.0	
Religion	X ± SD	0.98 ± 1.07	0.8 ± 0.88	*p* = 0.475
	Me	0.75	0.5	
	quartiles	0.0–2.0	0.0–1.5	
Use of emotional support	X ± SD	1.41 ± 0.98	1.77 ± 0.72	*p* = 0.016
	Me	1.25	2.0	
	quartiles	1.0–2.0	1.5–2.0	
Use of instrumental support	X ± SD	1.48 ± 0.76	1.78 ± 0.73	*p* = 0.008
	Me	1.5	2.0	
	quartiles	1.0–2.0	1.0–2.0	
Self-distraction	X ± SD	1.73 ± 0.76	1.81 ± 0.66	*p* = 0.788
	Me	1.75	2.0	
	quartiles	1.5–2.12	1.5–2.0	
Denial	X ± SD	1.14 ± 0.94	0.98 ± 0.88	*p* = 0.297
	Me	1.0	1.0	
	quartiles	0.38–1.5	0.0–1.5	
Venting	X ± SD	1.27 ± 0.87	1.26 ± 0.69	*p* = 0.787
	Me	1.25	1.25	
	quartiles	0.5–2.0	0.88–1.5	
Substance use	X ± SD	0.9 ± 1.05	0.73 ± 0.9	*p* = 0.481
	Me	0.5	0.5	
	quartiles	0.0–2.0	0.0–1.0	
Behavioral disengagement	X ± SD	1.07 ± 0.84	0.61 ± 0.73	*p* = 0.001
	Me	1.0	0.5	
	quartiles	0.5–2.0	0.0–1.5	
Self-blame	X ± SD	1.68 ± 0.88	1.35 ± 0.76	*p* = 0.029
	Me	1.5	1.5	
	quartiles	1.0–2.5	1.0–2.0	

n—number of subjects; X—mean; SD—standard deviation; Me—median; *p*—statistical value; * Mann–Whitney *U*-test. Source: author’s own analysis.

## Data Availability

Data sharing not applicable.
